# Latency period of lung cancer in relation to tobacco smoking in Korea

**DOI:** 10.4178/epih.e2026014

**Published:** 2026-03-30

**Authors:** Thi Tra Bui, Hee-Yeon Kang, Eunjung Park, Jin-Kyoung Oh

**Affiliations:** 1 Department of Public Health & AI, National Cancer Center Graduate School of Cancer Science and Policy, Goyang, Korea; 2 Division of Cancer Prevention, National Cancer Control Institute, National Cancer Center, Goyang, Korea

**Keywords:** Health policy, Lung neoplasms, Smoking, Time factors, Tobacco control

## Abstract

**OBJECTIVES:**

This mixed-methods observational study aimed to examine the temporal relationship between trends in cigarette smoking prevalence and lung cancer mortality and incidence rates, and to estimate the latency period between smoking initiation and lung cancer diagnosis among smokers at the individual level.

**METHODS:**

Smoking prevalence data for 1960–2022 were reconstructed using data from the Korea National Health and Nutrition Examination Survey (1998–2022). Lung cancer mortality data (1983–2022) and incidence data (1999–2022) were obtained from Statistics Korea and the Korea Central Cancer Registry. The population latency period was estimated using peak comparison and distributed lag non-linear models. The individual latency period was estimated as the average time interval between age at smoking initiation and age at lung cancer diagnosis using individual-level data from the National Health Insurance Service cohort.

**RESULTS:**

In men, smoking prevalence peaked around 1985, whereas lung cancer mortality peaked around 2000 during 1960–2022, indicating a lag time of approximately 15 years. In women, lung cancer mortality peaked in 2002, while smoking prevalence peaked around the same time. The population-level latency period between smoking and lung cancer incidence was estimated to be 15 years after adjustment for age, gender, and year of outcome. The individual latency period was estimated to be 42.6 years (standard deviation [SD], 12.5) in men and 34.4 years (SD, 14.2) in women. Smoking intensity and age at initiation did not appear to shorten the individual latency period.

**CONCLUSIONS:**

Estimates of the smoking-attributable lung cancer burden should account for historical smoking prevalence from approximately 15 years earlier.

## GRAPHICAL ABSTRACT

[Fig f4-epih-48-e2026014]

## Key Message

Smoking prevalence and lung cancer mortality and incidence rates in Korea showed clear temporal trends, particularly among men, with an approximate 15-year lag at the population level. Individual-level analyses indicated much longer latency periods from smoking initiation to lung cancer diagnosis over 40 years in men and over 30 years in women. These findings suggest that assessments of the smoking-attributable lung cancer burden on population health should incorporate historical smoking patterns from at least 15 years earlier.

## INTRODUCTION

Cigarette smoking remains a major public health concern. The total number of smokers has increased globally, from 0.99 billion in 1990 to 1.14 billion in 2019, primarily because of population growth [1]. Nevertheless, global cigarette smoking prevalence has declined substantially over the past 3 decades, decreasing by 27.5% among men and 37.7% among women since 1990 [1]. In Korea, the daily smoking rate among individuals aged 15 years and older is comparable to that of the Organization for Economic Cooperation and Development (15.4 vs. 16.0% in 2021) [2]. Notably, Korea exhibits a substantial gender disparity in smoking rates, with a prevalence of 26.3% among men compared with 4.5% among women [2].

Tobacco smoking is a well-established cause of lung cancer and is responsible for approximately 85% of cases [3]. Lung cancer is the leading cause of cancer-related death and has a low survival rate, even in high-income countries [4]. Globally, an estimated 1.8 million deaths from lung cancer occurred in 2020, accounting for approximately one-fifth of all cancer mortality [5]. In Korea, lung cancer has been the leading cause of cancer death since 2000 [6]. In 2022, it was the most commonly diagnosed cancer among men and the fourth most commonly diagnosed cancer among women [7].

A deeper understanding of temporal changes in tobacco smoking and lung cancer incidence and mortality is essential for evaluating current efforts and shaping future tobacco control policies. Although substantial progress has been made in reducing premature lung cancer deaths through tobacco control policies and smoking cessation campaigns in many countries, these reductions are not universal [8]. National smoking histories are key determinants of the current magnitude of lung cancer within a population [9]. As tobacco use declines, lung cancer incidence/mortality rates are expected to peak after a latency period—estimated at approximately 30 years in the United States [10]. Estimating the disease burden of tobacco smoking on population health requires accurate measurement of exposure [11]. However, the latency period has not been well studied in Korea, and findings from the United States or other Western countries may not be directly applicable because of differences in social and cultural contexts as well as tobacco epidemic patterns and control policies. Individual latency, defined as the time interval between smoking initiation and lung cancer diagnosis, is also of considerable public health interest [12]. It is likely to be influenced by biological processes, personal behaviors, and genetic and environmental factors. In this study, we aimed to examine the temporal relationship between trends in cigarette smoking prevalence and lung cancer mortality and incidence rates to infer the population latency period, and to use cohort data to estimate the individual latency period for lung cancer among current smokers.

## MATERIALS AND METHODS

This study examined time trends in smoking prevalence from 1960 to 2022, lung cancer mortality rates from 1983 to 2022, and lung cancer incidence rates from 1999 to 2022. Given the limited availability of data on historical smoking rates, we reconstructed annual smoking rates for 1960–2022 based on the Korea National Health and Nutrition Examination Survey (KNHANES) from 1998 to 2022 using an approach validated in previous research [13,14]. KNHANES is a nationwide, population-based, cross-sectional survey conducted annually among approximately 10,000 individuals aged 1 year and older, with a response rate of about 75% [15]. Age-specific and gender-specific smoking prevalence among adults aged ≥20 years was estimated using self-reported smoking status, age at smoking initiation, and smoking duration. Current smokers were defined as individuals who had smoked at least 5 packs of cigarettes (100 cigarettes) in their lifetime and were currently smoking every day or some days. They were classified as smokers from the year they began smoking through the survey year and as non-smokers before smoking initiation. Former smokers were classified as smokers from the year they initiated smoking until the year they quit, derived from reported smoking duration, and as non-smokers before initiation and after cessation. Participants who reported never smoking were classified as non-smokers for each preceding year in which they met the age criteria through the survey year. Crude smoking rates and smoking rates by 5-year age group for men and women were estimated annually from 1960 to 2022 using pooled data from all survey years, with sampling weights applied to account for the complex survey design. Crude smoking rates were used instead of age-standardized rates because older age groups were increasingly underrepresented in earlier years. Age-specific and gender-specific rates of lung cancer incidence and mortality, along with the number of cases, were obtained from the Korea Central Cancer Registry and Statistics Korea [16,17]. Lung cancer incidence by histological type focused on squamous cell carcinoma and adenocarcinoma—the 2 most common types of lung cancer, which have different carcinogenic mechanisms. Age-standardized rates of lung cancer incidence and mortality among men and women were estimated using the 2000 Korean population as the standard [18]. The population latency period was first examined visually by comparing the timing of peaks in smoking prevalence with those in lung cancer incidence/mortality [19]. In addition, we applied distributed lag non-linear models (DLNMs) to estimate the lag time associated with the strongest relationship between smoking prevalence and lung cancer incidence [20]. This approach allows the modeling of delayed health effects of prolonged exposures with varying intensities by using a cross-basis that combines 2 functions: one for the exposure-response relationship and another for the lag structure [21]. As a compromise between the maximum lag duration and the number of data points available for lung cancer incidence, we examined lag times ranging from 0 years to 30 years. Because reconstructed smoking rates were available for 1980–2022, which better represented older age groups, lung cancer incidence data from 2010 to 2022 were analyzed. Historical smoking prevalence in 5-year age groups (20–24, 25–29, …, 80–84, and ≥85 years) was matched to lung cancer incidence in the corresponding 5-year age groups (40–44, …, 80–84, and ≥85 years), as appropriate. For example, lung cancer incidence in the 40–44-year age group in 2022 was matched with smoking prevalence in the same age group in 2022 (lag=0), in 2021 through 2018 (lag=1 to 4), and in the 35–39-year age group in 2017 (lag=5). DLNMs were fitted using the following specifications: lung cancer incidence was assumed to follow a negative binomial distribution, and non-linearity in both the exposure and lag dimensions was modeled using restricted cubic splines. Analyses were conducted by demographic strata defined by gender, age group, and outcome year. Population-at-risk estimates for lung cancer incidence between 2010 and 2022 were obtained from the Korean Statistical Information Service. Full model details are described elsewhere [20].

At the individual level, the latency period of lung cancer in relation to tobacco smoking was defined as the interval between smoking initiation and lung cancer diagnosis. We used a customized sample of the National Health Insurance Service (NHIS) cohort that included 13,034,526 individuals aged 20 years and older who underwent the nationwide general health examination in 2006–2007 and were cancer-free during that period. To use information on smoking duration available since 2009, we included participants who attended the general health examination in 2009–2010 (n=9,882,140). Among them, 92,145 participants were identified as having lung cancer between 2009 and 2022. Lung cancer diagnoses were identified using International Classification of Diseases, 10th revision codes C33–C34 and were further confirmed using the special code “V193,” which was introduced to expand insurance benefits for patients with cancer. We excluded 10,350 cases diagnosed during 2009–2010 to eliminate prevalent cases and 357 patients with missing information on age, gender, or smoking status. The final study population included 81,438 patients with lung cancer (56,932 men and 24,506 women). Among these, 29,904 patients (28,823 men and 1,081 women) who reported being current smokers and had baseline data on smoking duration were analyzed to estimate the individual latency period. The selection of study participants is presented in Supplementary Material 1. Participants were asked about their lifetime smoking status, smoking duration up to the time of examination, and average number of cigarettes smoked per day. Because direct information on age at smoking initiation was not available, we derived this variable from smoking duration, defined as the period from the year of smoking initiation to either the examination year or smoking cessation, under the assumption that smoking was continuous (i.e., with no multiple cessation periods). However, because data on duration since quitting were unavailable, age at smoking initiation could be derived only for current smokers by subtracting smoking duration from age at examination. An illustration of how age at smoking initiation was derived from smoking duration for ever-smokers is provided in Supplementary Material 2. The individual latency period was then estimated as the interval between age at smoking initiation and age at lung cancer diagnosis.

### Statistical analysis

Information on the number of cigarettes smoked per day was also collected to examine the dose-related association with age at lung cancer diagnosis and individual latency period. Analyses were conducted using SAS Enterprise Guide 7.1 (SAS Institute Inc., Cary, NC, USA). DLNMs were analyzed using the “dlnm” package in R version 4.5.2 (R Foundation for Statistical Computing, Vienna, Austria).

### Ethics statement

This study used anonymized secondary data and was exempted from review by the Institutional Review Board of the National Cancer Center, Korea (NCC2023-0114). The requirement for informed consent was waived for the same reason.

## RESULTS

### Trends in smoking prevalence and lung cancer incidence and mortality rates

**Figure 1.**
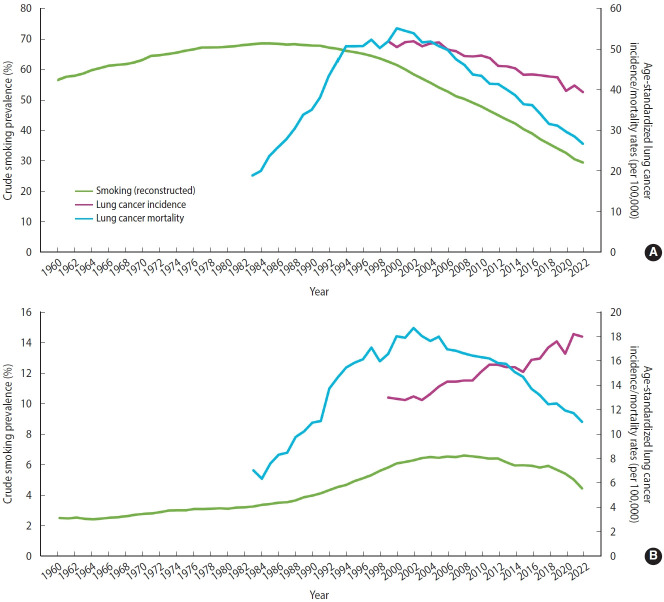
Cigarette smoking prevalence and lung cancer incidence and mortality rates in Korea during 1960−2022 among men (A) and women (B).

Figure 1 presents reconstructed smoking prevalence alongside lung cancer incidence and mortality rates by gender between 1960 and 2022. Among men (Figure 1A), smoking prevalence increased steadily from 57% in 1960 and peaked at 68% around 1984–1985 before declining thereafter. Lung cancer incidence decreased continuously from 1999 to 2022, with the highest age-standardized rate recorded in 1999 at 52 per 100,000. Notably, lung cancer mortality rose sharply, peaked at 55 per 100,000 in 2000, and then declined consistently. The time difference between the peak in smoking prevalence and the peak in lung cancer mortality was approximately 15 years. Among women (Figure 1B), smoking prevalence peaked more recently, reaching approximately 7% in 2008. While lung cancer mortality rates showed a pattern similar to that observed in men, lung cancer incidence in women increased steadily over time, in contrast to the decreasing trend in men. The peaks in smoking prevalence and lung cancer mortality appeared to occur at around the same time. By histological type (Supplementary Material 3), the age-standardized incidence rate of squamous cell carcinoma declined among men, whereas that of adenocarcinoma—the more common type among women—showed an upward trend.

**Figure 2.**
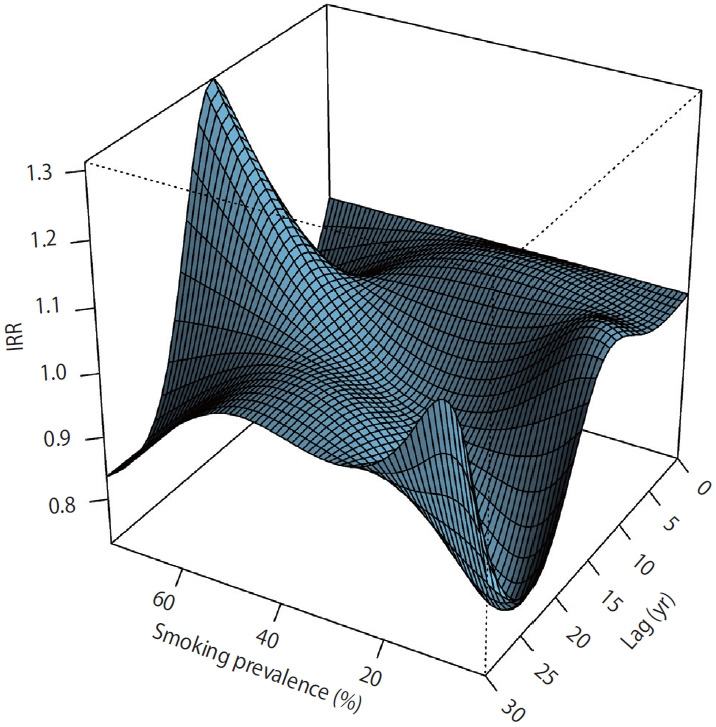
Two-dimensional exposure–lag response surface illustrating the combined effects of lag time and smoking prevalence on the predicted incidence rate ratio (IRR) of lung cancer.

In the DLNM analyses (Figure 2), among the lag times examined from 0 years to 30 years, a lag of 15 years showed the highest predicted incidence rate ratio (1.31; 95% confidence interval, 1.16 to 1.49) for the association between smoking prevalence and lung cancer incidence at a smoking prevalence of 75%.

### Individual latency period of lung cancer in relation to cigarette smoking

Among patients with lung cancer, the median age at diagnosis was 71 years (interquartile range [IQR], 64–77) in men and 70 years (IQR, 62–77) in women (Table 1). At baseline, never smokers accounted for 22.4% of men and 94.1% of women. Among men, current smokers were more likely to be diagnosed with lung cancer at a younger age than never smokers (median age, 70 [63, 76] vs. 73 [65, 79] years). In contrast, among women, current smokers tended to be diagnosed at an older age than never smokers (73 [66, 80] years vs. 69 [62, 76] years).

**Figure 3.**
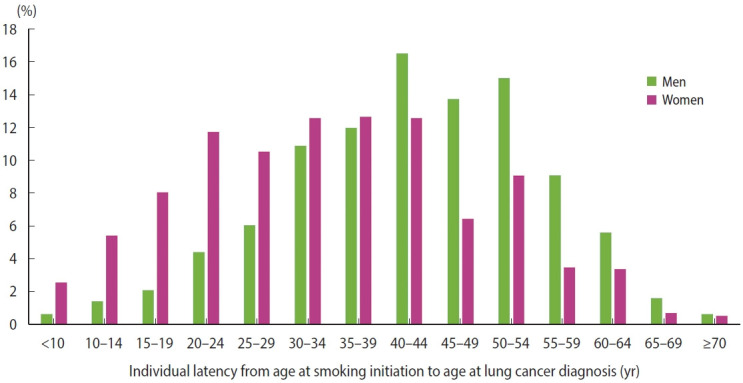
Distribution of individual latency periods among lung cancer patients who reported current smoking at baseline (based on National Insurance Health Service data).

In the subgroup of current smokers (Table 2), the latency period was estimated to be 42.6 years (standard deviation [SD], 12.5) in men and 34.4 years (SD, 14.2) in women. Men began smoking at a median age of 24 years (IQR, 20–31), whereas women tended to begin smoking at an older age (38 years [IQR, 30–46]). Individuals who started smoking earlier were more likely to be diagnosed with lung cancer at a younger age than those who started later. This pattern was observed in both men (median age, 69 [62, 76] vs. 72 [66, 77] years, p<0.001) and women (72 [64, 79] vs. 74 [66, 80] years, p<0.001). In addition, those who smoked more heavily (>20 cigarettes/day) tended to be diagnosed at a younger age than those who smoked 1–10 cigarettes/day (men: 66 [61, 72] vs. 74 [68, 79] years, p<0.001; women: 71 [60, 76] vs. 75 [67, 81] years, p<0.001). However, earlier smoking initiation and higher smoking intensity did not appear to shorten the latency interval. The distributions of age at smoking initiation and age at lung cancer diagnosis among current smokers are presented in Supplementary Material 4. The distribution of individual latency periods is shown in Figure 3.

## DISCUSSION

This study examined trends in smoking prevalence and lung cancer incidence and mortality in Korea from 1960 to 2022 and identified a 15-year lag between smoking prevalence and lung cancer incidence/mortality rates at the population level. At the individual level, the estimated latency period between smoking initiation and lung cancer diagnosis was approximately 43 years for men and 34 years for women.

Between 1960 and 2022, the tobacco epidemic transition in Korea broadly aligned with the 4-stage model proposed by Lopez et al. [9], although some deviations were observed. By 1985, Korea had moved beyond Stage I, which is characterized by low smoking prevalence in both men and women and a rapid rise among men. Instead, the high smoking prevalence among men around 1985 (approximately 70%) indicates that Korea was in the latter half of Stage II, during which men smoking rates had peaked and begun to decline. Stage III (1985–2005) was marked by a continued decline in smoking prevalence and a peak in lung cancer mortality among men. Aligned with the model, smoking trends among women lagged behind those among men by 2 decades—that is, plateauing, peaking, and then declining during this stage. In Stage IV (from 2005 onward), smoking rates are expected to decline steadily in both genders. However, in Korea, women smoking prevalence remained stable and even showed a slight increase, particularly among women in their 20s and 30s, likely because of changing socioeconomic and cultural factors, as observed in previous research [22,23].

In our study, lung cancer incidence by subtype showed distinct patterns consistent with smoking-related risk. Squamous cell carcinoma exhibited trends similar to those of smoking prevalence in both men and women, with a steady decline over time. In contrast, adenocarcinoma continued to increase, contributing to the rising overall incidence of lung cancer among women. These patterns highlight the predominant role of smoking as a risk factor for lung cancer incidence in men, whereas other factors, such as occupational exposures, may play a more substantial role among women.

In this study, the estimated age at smoking initiation was older than that reported in previous studies in Korea [24] and the United States [25]. A previous study using KNHANES data found that smokers began smoking at around 19 years of age for men and 25 years of age for women (2007) [24]. Using NHIS data, age at smoking initiation was inferred from smoking duration among current smokers, which may reflect the age at onset of regular smoking rather than that of initial experimentation. Earlier smoking initiation and heavier smoking were associated with a younger age at lung cancer diagnosis. However, individuals who began smoking earlier tended to have a longer latency period, possibly because earlier initiation extends the overall duration of exposure rather than shortening the time to lung cancer diagnosis. In addition, these effects may be overshadowed by the stronger influence of age, involving reduced ability to defend against inhaled carcinogens or the cumulative effects of deposits in the respiratory tract [26].

Among women, current smokers were diagnosed at an older age than never smokers, which may reflect residual confounding rather than a true protective effect. Potential confounders include secondhand smoke exposure, occupational exposures (e.g., kitchen fumes among cooks), family history of lung cancer, and comorbid chronic obstructive pulmonary disease [26-28]. A previous study suggested that the curves for smoking prevalence and lung cancer mortality appeared later in women than in men [11]. However, our study observed similar patterns of lung cancer mortality in both genders, suggesting a substantial contribution of secondhand smoke exposure to the lung cancer burden among Korean women, given the high proportion of never smokers in this population. Korea implemented a ban on smoking in public places in 2012; however, the MPOWER component “Protect people from tobacco smoke” continued to be rated weak until recently [22]. In addition, underreporting and cultural reporting bias may help explain the high proportion of never smokers in this population [29].

Lung cancer progresses through multiple stages: initiation, promotion, and malignant conversion [30]. Smoking can influence any of these stages; however, smoking-related promotion is considered the most critical in lung carcinogenesis because of the clonal expansion of mutated cells, which contributes to the rationale for latency [30]. Unlike acute exposures, for which time since exposure is well defined and often strongly influences disease risk, extended exposures present a challenge: the definition of time since exposure and its association with disease outcomes become less clear [31]. Estimates of the latency period for lung cancer vary across studies, reflecting differences in definitions and methodological approaches. A longitudinal study estimated the population latency period—defined as the interval between population averages of cigarette consumption (assumed to begin at age 20 years) and lung cancer mortality—to range from approximately 10 years to 30 years [26]. In the United States, lung cancer rates peaked around 1990, approximately 10–20 years after the peak in national cigarette sales [26]. In Spain, a study using a cross-correlation approach with reconstructed smoking data reported population latency periods ranging from 20 years to 34 years for men and 10 years to 37 years for women [14]. However, cross-correlation does not appear to be an appropriate approach for our data without substantial preprocessing because the temporal patterns of smoking and lung cancer incidence/mortality differ in duration and in their rates of change over time. Other studies in Spain using distributed lag non-linear models estimated a 6-year lag [20] and a 15-year lag [32] after controlling for gender, age, outcome year, and population at risk. In our study, both the comparison of peak timing between smoking and lung cancer and the statistical analyses yielded consistent results, supporting the robustness of our findings. Therefore, we suggest that historical smoking prevalence from approximately 15 years earlier provides a reasonable basis for estimating the current smoking-attributable disease burden. At the individual level, a long latency period of 30–40 years may hinder public awareness of the substantial health hazards of tobacco use [9]. Nevertheless, smokers may exhibit measurable declines in lung function as early as 25–30 years of age, with pathologically definable pulmonary emphysema typically developing in their 50s [33]. In contrast, those who quit smoking, especially at younger ages, can largely eliminate the increased risk of cancer-related death associated with continued smoking [34].

Historically, anti-smoking health policies have not been promoted as aggressively in Korea as in some other countries [23]. Smoking prevalence began to decline in the late 1980s, possibly because of the introduction of smoke-free policies during the 1988 Olympic Games [35]. However, it was not until 1995—3 decades after the United States—that Korea implemented national tobacco control measures through enactment of the National Health Promotion Act [36]. In 2005, Korea ratified the Framework Convention on Tobacco Control [22]. Since then, multiple anti-smoking regulations have been introduced, including increases in cigarette prices, higher taxation of tobacco products, implementation of smoke-free public spaces, a ban on tobacco advertisements in stores, and graphic health warnings on packaging [22,23]. Despite these efforts, progress toward a smoke-free population has been slower in Korea than in countries such as the United States [2] and Singapore [9]. Further improvements are needed, particularly in raising tobacco prices, implementing a total ban on smoking in indoor and public places, regulating tobacco advertising, and enforcing workplace smoking policies [22,23].

This study has several limitations. First, data on smoking prevalence in Korea before the 1980s are limited. Use of reconstructed smoking prevalence may have led to overestimation because earlier years are likely to over-represent younger age groups, who tend to smoke more than older adults. However, a smoking prevalence of 84.8% was reported among men in 1980 [37], suggesting that the peak prevalence of 68% among men observed in the mid-1980s in our study is plausible. Second, measurements of urinary cotinine concentrations suggest that self-reported smoking status in the Korean population tends to be underestimated, especially among women [29]. Third, estimating age at smoking initiation from smoking duration may have introduced bias into the individual latency-period estimate. Specifically, an earlier estimated age at smoking initiation (i.e., longer smoking duration) would lengthen the individual latency period, whereas a later estimated age at smoking initiation (i.e., shorter smoking duration) would shorten it. However, previous research reported that more than 80% of the Korean population began smoking before 20 years of age [38]. This suggests that variability in age at smoking initiation is relatively limited and that its impact on our individual latency estimates is therefore likely to be minimal. In addition, we were unable to estimate the latency period among former smokers because of the lack of data on duration since quitting. Because former smokers were not included in the analyses, our findings are subject to selection bias. Furthermore, smoking behavior tends to change over time, including relapse and cessation. However, because the harmful effects of smoking are likely to accumulate, only long-term changes may reduce the risk of lung cancer associated with tobacco smoking. Therefore, the individual latency period estimated in this study is likely to reflect the interval from smoking initiation to lung cancer diagnosis averaged across the Korean population, including both persistent smokers and quitters, based on smoking information collected at a cross-sectional time point (i.e., at baseline in our study). Furthermore, factors beyond tobacco smoking may influence lung cancer risk, including occupational and environmental exposures, which may contribute differently to the etiologies of various histological types. Our findings primarily reflect changes in tobacco smoking, a major contributor to squamous cell carcinoma, which is more common in men. Interpretation of the results in women should be cautious, as other risk factors may play a greater role in adenocarcinoma, the predominant type among women. Due to the lack of lung cancer incidence data during the 1980s–1990s, the estimation of the population latency period using the peak comparison approach was based on lung cancer mortality rates to approximate incidence rates, given the high fatality of this disease. Moreover, because of the ecological nature of our population-based latency estimation, we were unable to distinguish lung cancer cases occurring in non-smokers, which is particularly relevant when considering the effects of other factors [26]. Nonetheless, their impact is generally considered much less important. Given that it takes about 12 years or more after smoking cessation to halve the risk observed in continuing smokers [39], the rising proportion of former smokers, as current smoking rates decline, may slow the reduction in lung cancer mortality. Finally, because our results are based on data from the Korean population, they may not be generalizable to other populations. Given these limitations, the findings should be interpreted with caution. Further research in diverse settings would be valuable for deepening our understanding of the latency period between tobacco smoking and lung cancer development.

In conclusion, it may take approximately 15 years after a change in smoking prevalence for a corresponding change in lung cancer incidence/mortality rates to be observed in the Korean population. At the individual level, the interval between smoking initiation and lung cancer diagnosis was estimated to be 43 years for men and 34 years for women. Estimates of the smoking-attributable lung cancer burden should take historical smoking prevalence from 15 years earlier into account.

## Figures and Tables

**Figure f4-epih-48-e2026014:**
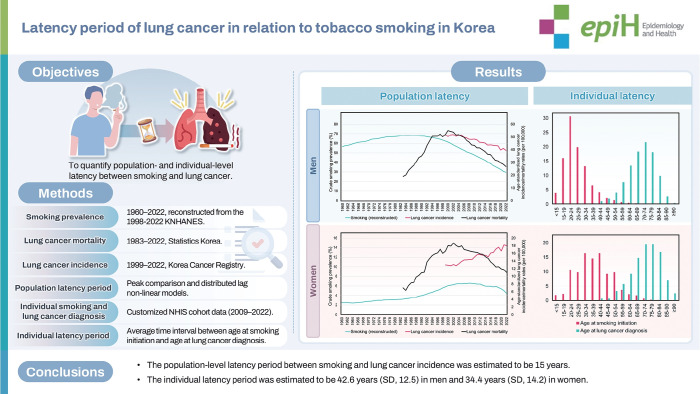


**Table 1.** General characteristics of lung cancer patients at baseline (2009−2010) (based on National Health Insurance Service data)

Characteristics	Men (n=56,932)	Women (n=24,506)
Age at baseline (yr)		
<40	1,253 (2.2)	539 (2.2)
40−49	4,517 (7.9)	2,657 (10.8)
50−59	13,201 (23.2)	7,017 (28.6)
60−69	21,266 (37.4)	8,033 (32.8)
70−79	15,020 (26.4)	5,556 (22.7)
≥80	1,675 (2.9)	704 (2.9)
Mean±SD	62.93±10.10	61.29±10.58
Smoking status		
Never smokers	12,756 (22.4)	23,054 (94.1)
Former smokers	14,960 (26.3)	341 (1.4)
Current smokers	29,216 (51.3)	1,111 (4.5)
Age at lung cancer diagnosis (yr)		
Overall	71 [64, 77]	70 [62, 77]
Never smokers	73 [65, 79]	69 [62, 76]
Former smokers	72 [65, 78]	73 [63, 81]
Current smokers	70 [63, 76]	73 [66, 80]

Values are presented as number (%) or median [first quartile, third quartile]. SD, standard deviation.

**Table 2.** Individual latency periods among lung cancer patients in the current smoking subgroup (based on National Insurance Health Service cohort data)

Characteristics	Men (n=28,823)	Women (n=1,081)
Age at smoking initiation, n (%)		
<15	1,141 (4.0)	20 (1.9)
15−19	4,626 (16.0)	25 (2.3)
20−29	14,572 (50.6)	221 (20.4)
30−39	5,724 (19.9)	335 (31.0)
40−49	1,826 (6.3)	278 (25.7)
50−59	645 (2.2)	146 (13.5)
≥60	289 (1.0)	56 (5.2)
Median [Q1, Q3]	24 [20, 31]	38 [30, 46]
Age at lung cancer diagnosis, median [Q1, Q3]		
Overall	70 [63, 76]	74 [66, 80]
By age at smoking initiation		
<30	69 [62, 76]	72 [64, 79]
≥30	72 [66, 77]	74 [66, 80]
p-value^1^	<0.001	<0.001
By smoking levels (cigarettes/day)		
1−10	74 [68, 79]	75 [67, 81]
11−20	69 [63, 75]	71 [65.5, 78]
>20	66 [61, 72]	71 [60, 76]
p-value^2^	<0.001	<0.001
Individual latency period, mean±SD		
Overall	42.62±12.49	34.42±14.24
By age at smoking initiation		
<30	46.64±10.83	47.66±12.54
≥30	33.01±10.82	30.10±11.92
p-value^1^	<0.001	<0.001
By smoking levels (cigarettes/day)		
1−10	42.90±14.50	33.74±14.71
11−20	42.55±11.80	35.75±13.03
>20	42.85±10.43	38.26±11.05
p-value^2^	<0.001	<0.001

SD, standard deviation; Q1, first quartile; Q3, third quartile.

1 Student’s *t*-test.

2 One-way analysis of variance.

## Data Availability

The data used in this study (NHIS-2024-1-232) were provided by the National Health Insurance Service. It is available for researchers who meet the criteria for access to confidential data. To protect personal information, the data cannot be shared because NHIS prohibits the transfer, rental, or sale of the database to third parties except for researchers who have been approved for access. The NHIS data can be requested through its website (https://nhiss.nhis.or.kr). Further information is available from the corresponding author upon request.

## Supplementary materials

Supplementary Material 1. Study flowchart.

Supplementary Material 2. Derivation of age at smoking initiation using smoking duration among ever-smokers.

Supplementary Material 3. Smoking prevalence and lung cancer incidence rates by histological type (squamous cell carcinoma and adenocarcinoma) in Korea during 1960−2022 among men (A) and women (B).

Supplementary Material 4. Distribution of age at smoking initiation and age at lung cancer diagnosis among men (A) and women (B) (based on National Insurance Health Service data).
