# Supplementary material for: Latency period of lung cancer in relation to tobacco smoking in Korea
Source: Epidemiol Health. 2026 Mar 30;48:e2026014. doi: 10.4178/epih.e2026014 (PMC13219974; doi:10.4178/epih.e2026014)
Supplement: Supplementary Material 1. — Study flowchart. [file epih-48-e2026014-Supplementary-1.ppt]

## Slide 1
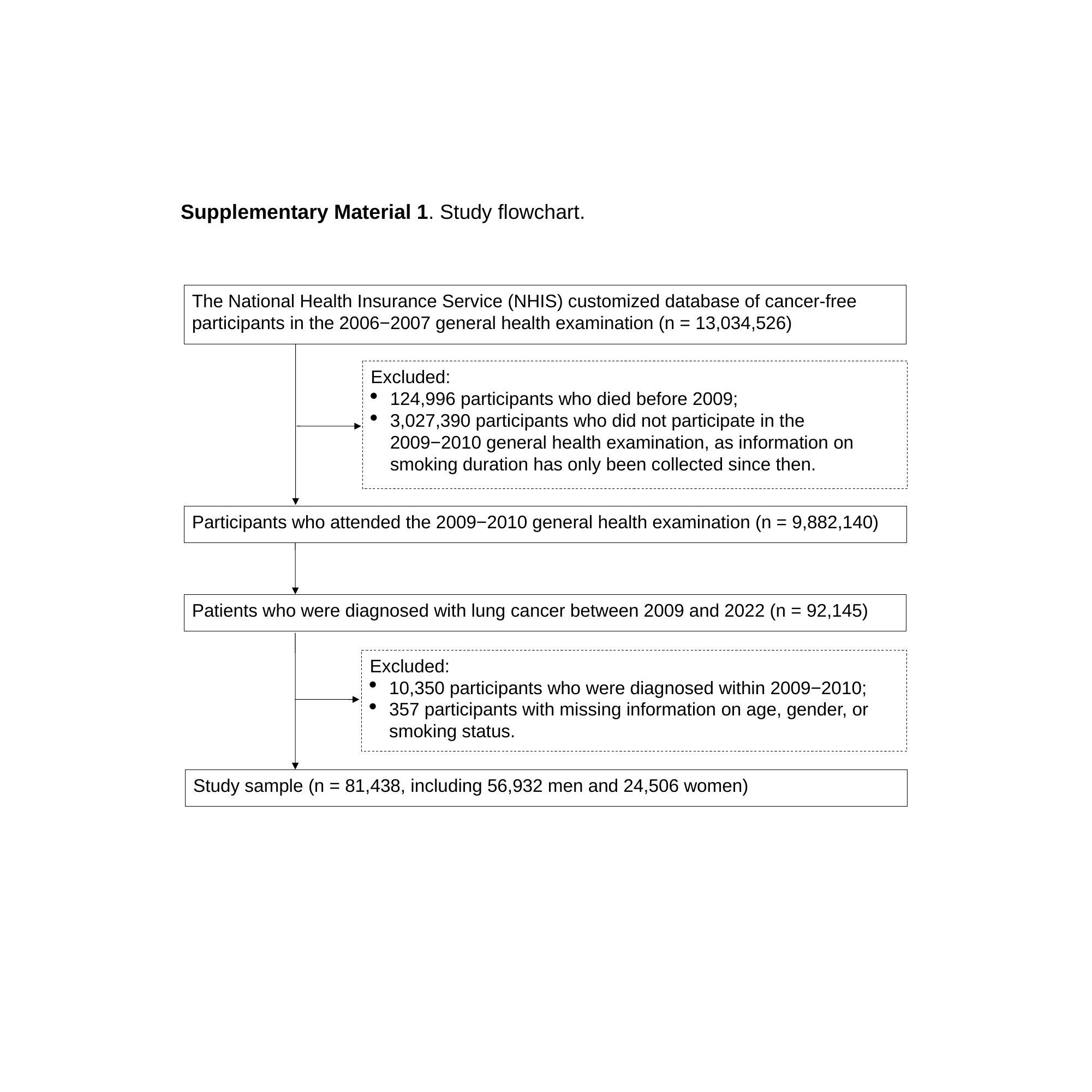

Supplementary Material 1. Study flowchart.
The National Health Insurance Service (NHIS) customized database of cancer-free participants in the 2006−2007 general health examination (n = 13,034,526)
Excluded:
124,996 participants who died before 2009;
3,027,390 participants who did not participate in the 2009−2010 general health examination, as information on smoking duration has only been collected since then.
Participants who attended the 2009−2010 general health examination (n = 9,882,140)
Patients who were diagnosed with lung cancer between 2009 and 2022 (n = 92,145)
Excluded:
10,350 participants who were diagnosed within 2009−2010;
357 participants with missing information on age, gender, or smoking status.
Study sample (n = 81,438, including 56,932 men and 24,506 women)
