# Supplementary material for: Latency period of lung cancer in relation to tobacco smoking in Korea
Source: Epidemiol Health. 2026 Mar 30;48:e2026014. doi: 10.4178/epih.e2026014 (PMC13219974; doi:10.4178/epih.e2026014)
Supplement: Supplementary Material 2. — Derivation of age at smoking initiation using smoking duration among ever-smokers. [file epih-48-e2026014-Supplementary-2.ppt]

## Slide 1
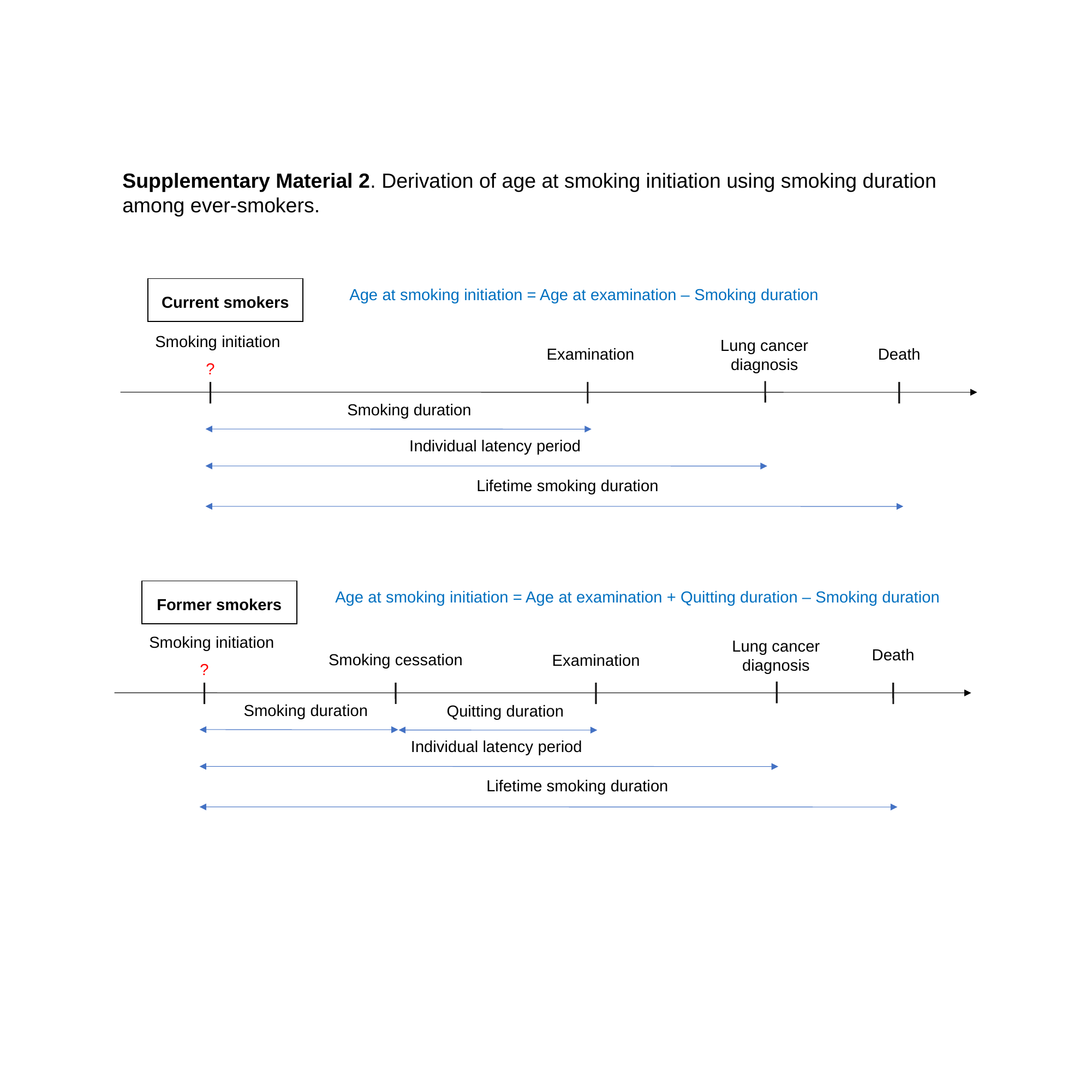

Supplementary Material 2. Derivation of age at smoking initiation using smoking duration among ever-smokers.
Current smokers
Age at smoking initiation = Age at examination – Smoking duration
Smoking initiation
Lung cancer diagnosis
Examination
Death
?
Smoking duration
Individual latency period
Lifetime smoking duration
Former smokers
Age at smoking initiation = Age at examination + Quitting duration – Smoking duration
Smoking initiation
Lung cancer diagnosis
Death
?
Examination
Smoking duration
Individual latency period
Lifetime smoking duration
Smoking cessation
Quitting duration
