# Supplementary material for: Latency period of lung cancer in relation to tobacco smoking in Korea
Source: Epidemiol Health. 2026 Mar 30;48:e2026014. doi: 10.4178/epih.e2026014 (PMC13219974; doi:10.4178/epih.e2026014)
Supplement: Supplementary Material 3. — Smoking prevalence and lung cancer incidence rates by histological type (squamous cell carcinoma and adenocarcinoma) in Korea during 1960−2022 among men (A) and women (B). [file epih-48-e2026014-Supplementary-3.ppt]

## Slide 1
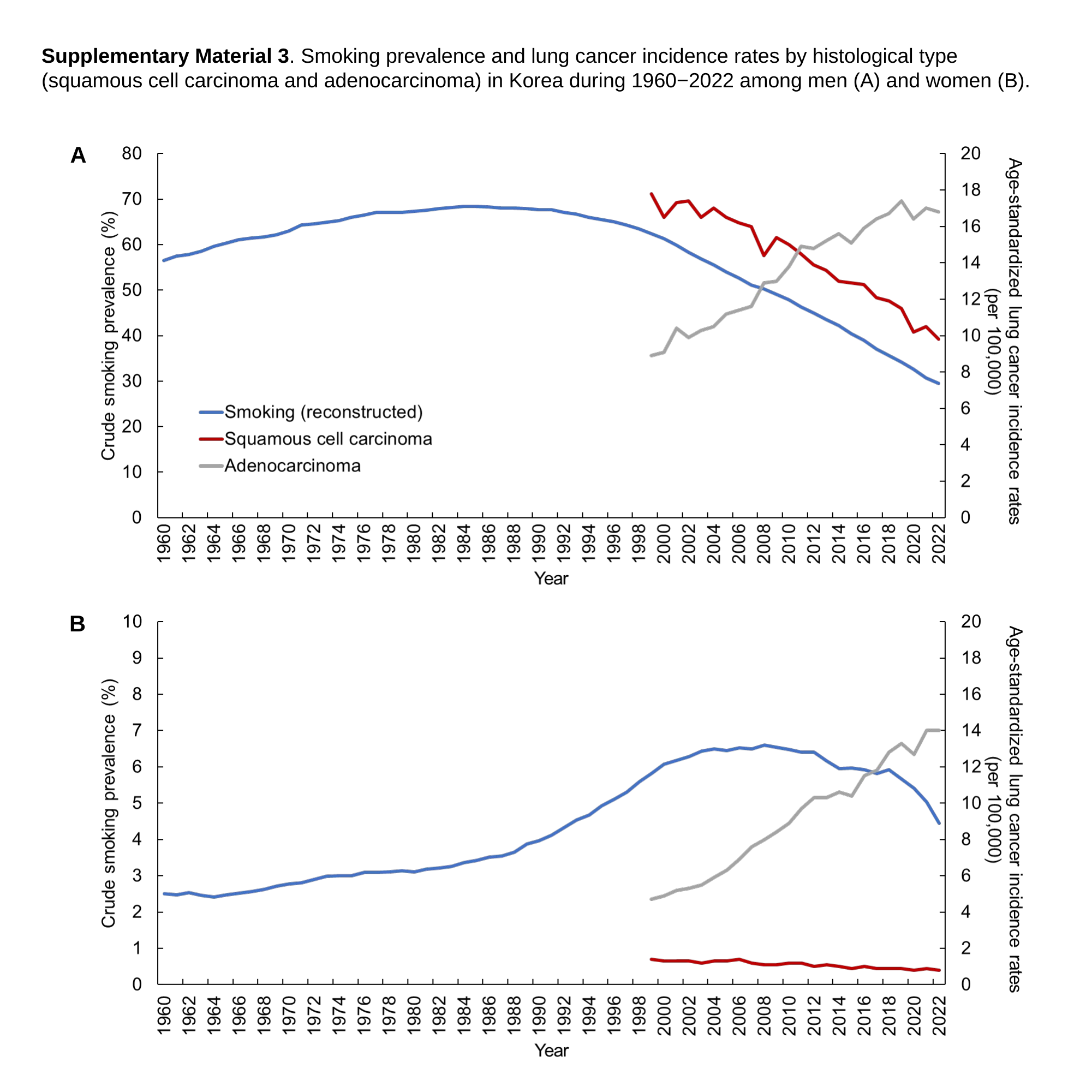

Supplementary Material 3. Smoking prevalence and lung cancer incidence rates by histological type (squamous cell carcinoma and adenocarcinoma) in Korea during 1960−2022 among men (A) and women (B).
A
B
