# Supplementary material for: Latency period of lung cancer in relation to tobacco smoking in Korea
Source: Epidemiol Health. 2026 Mar 30;48:e2026014. doi: 10.4178/epih.e2026014 (PMC13219974; doi:10.4178/epih.e2026014)
Supplement: Supplementary Material 4. — Distribution of age at smoking initiation and age at lung cancer diagnosis among men (A) and women (B) (based on National Insurance Health Service data). [file epih-48-e2026014-Supplementary-4.ppt]

## Slide 1
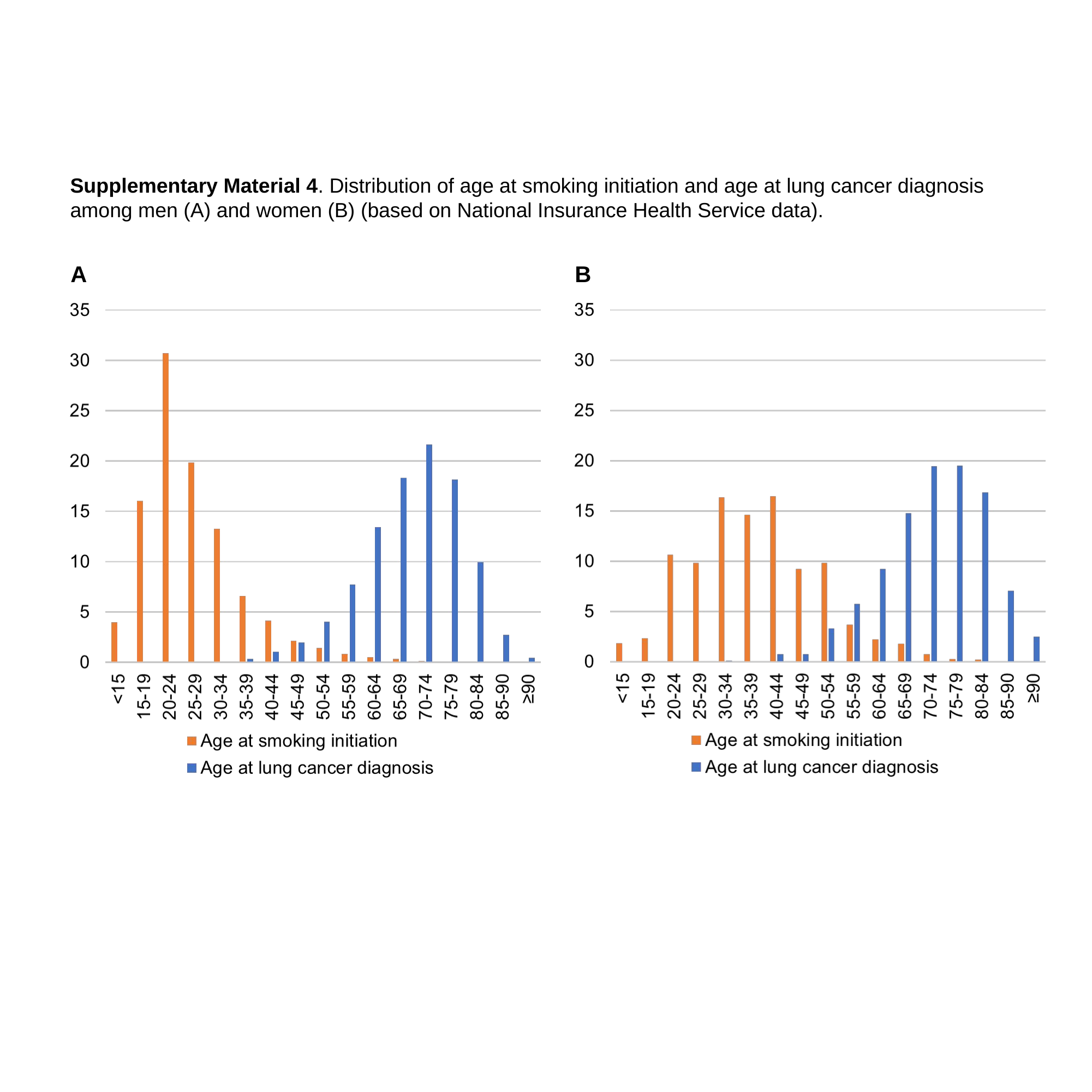

Supplementary Material 4. Distribution of age at smoking initiation and age at lung cancer diagnosis among men (A) and women (B) (based on National Insurance Health Service data).
A
B
